# Are vegetation-related roughness changes the cause of the recent decrease in dust emission from the Sahel?

**DOI:** 10.1002/grl.50273

**Published:** 2013-05-13

**Authors:** Sophie M Cowie, Peter Knippertz, John H Marsham

**Affiliations:** 1 Institute for Climate and Atmospheric Science, School of Earth and Environment, University of LeedsLeeds, UK, LS2 9JT; 2 Institute for Climate and Atmospheric Science, School of Earth and Environment, University of LeedsLeeds, UK, LS2 9JT; 3 National Centre for Atmospheric Science, School of Earth and Environment, University of LeedsLeeds, UK, LS2 9JT

**Keywords:** Sahel, dust emission, decadal trends, wind-speed, vegetation, roughness length

## Abstract

[1] Since the 1980s, a dramatic downward trend in North African dustiness and transport to the tropical Atlantic Ocean has been observed by different data sets and methods. The precise causes of this trend have previously been difficult to understand, partly due to the sparse observational record. Here we show that a decrease in surface wind speeds associated with increased roughness due to more vegetation in the Sahel is the most likely cause of the observed drop in dust emission. Associated changes in turbulence and evapotranspiration, and changes in large-scale circulation, are secondary contributors. Past work has tried to explain negative correlations between North African dust and precipitation through impacts on emission thresholds due to changes in soil moisture and vegetation cover. The use of novel diagnostic tools applied here to long-term surface observations suggests that this is not the dominating effect. Our results are consistent with a recently observed global decrease in surface wind speed, known as “stilling”, and demonstrate the importance of representing vegetation-related roughness changes in models. They also offer a new mechanism of how land-use change and agriculture can impact the Sahelian climate. **Citation**: Cowie, S. M., P. Knippertz, and J. H. Marsham (2013), Are vegetation-related roughness changes the cause of the recent decrease in dust emission from the Sahel?, Geophys. Res. Lett., 40, 1868–1872, doi:10.1002/grl.50273

## 1. Introduction

[2] North Africa is the world's largest dust source [*Goudie and Middleton*, 2001]. Dust emission from this region occurs from both the hyper-arid Sahara and the semi-arid Sahel, where summer rains allow a seasonal vegetation cover and agricultural activities. There is debate as to the relative importance of the two regions, which roughly straddle either side of 18°N latitude, to the total dust emission and export. The Sahara contains some of the most productive dust sources [*Engelstaedter and Washington*, 2007], although interannual and seasonal variability is mostly due to the sensitivity of the Sahel sources to rainfall, vegetation, and wind field changes [*Zender and Kwon*, 2005; *Moulin and Chiapello*, 2004; *Prospero and Lamb*, 2003; *Evan et al*., 2006]. Vegetation and rainfall have increased across the Sahel since the mid-1980s [*Olsson et al*., 2005; *Fensholt et al*., 2012]. At the same time, surface observations of visibility across northern Africa [*Mahowald et al*., 2007] and satellite estimates over the downstream tropical Atlantic [*Evan and Mukhopadhyay*, 2010] and Sahel [*Chiapello et al*., 2005] indicate a downward trend in dustiness from the early to mid-1980s to the present day. There are concurrent observations of weaker winds over the Sahel [*Mahowald et al*., 2007], but the mechanisms linking these trends have not been established.

[3] A general challenge in investigating the causes of this dust trend is the sparse observational record from source regions. Satellite-based data sets are short and mainly provide aerosol optical thickness but not emission directly [*Knippertz and Todd*, 2012]. Retrievals work best over oceans, where loadings are influenced by transport and deposition [*Evan et al*., 2006]. Over the Sahel, clouds, high column water vapor, and shallow dust layers hamper quantitative estimates [*Brindley et al*., 2012]. It is therefore desirable to make optimal use of existing long-term data from standard surface weather stations. Previous studies have used horizontal visibility [*Engelstaedter et al*., 2003; *Mbourou et al*., 1997], occurrence of suspended dust [*Klose et al*., 2010], and measurements of 10 m mean wind speed [*Mahowald et al*., 2007] to investigate dustiness over the Sahel. Very little use has been made of routine eye observations of dust emission at weather stations.

## 2. Data and Methods

**Figure 1:**
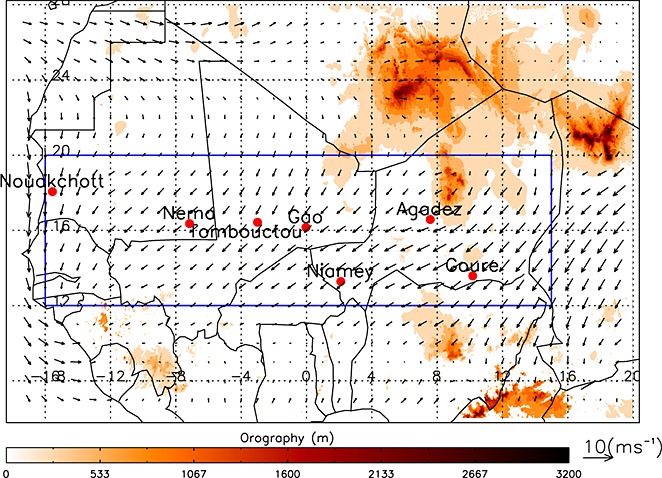
Map showing the location of the seven Sahelian stations used in this study (red dots with labels), the orography (shaded in m above mean sea level according to the legend), and the domain used for averaging ERA-Interim reanalysis data in blue. Winter mean (December–February) 10 m wind vectors from ERA-Interim are also included (scale in bottom right corner).

[4] We use the seven Sahelian stations of Nouakchott (World Meteorological Organization station number 61442), Nema (61497), Tombouctou (61223), Gao (61226), Niamey (61052), Agadez (61024), and Gouré (61045) (see Figure 1 for locations), which are part of the standard SYNOP surface observation network and typically report every 3 h. All seven stations are located in the relatively flat parts of the Sahel, away from the main mountain ranges (Figure 1). Wind speed observations are 10 min means, measured at 10 m height above ground. Reports of dust emission events, defined as the SYNOP “present weather” (ww) codes 7–9, 30–35, and 98 and representing dust emission of varying severities such as “Dust or sand raised by wind” (ww = 7) to “Severe duststorm or sandstorm” (ww = 33–35), were used to investigate dust emission (similar to *Ackerman and Cox* [1989]). We defined the parameter “frequency of dust emission events” (*FDE*) as the fraction of all reports containing these ww codes. We purposely omitted the frequently reported ww code 6 (“dust suspended, but not raised near the station”) to exclude transport events. The seven stations were selected on the basis of a minimum of 1000 dust observations overall during the time period 1984–2010 and at least 500 observations per year for each of the 27 years.

[5] In addition, 6-hourly 10 m *u* and *v* wind vectors from the European Centre for Medium-Range Weather Forecasts ERA-Interim reanalysis at a horizontal resolution of 80 km were used for the area inside the blue box shown in Figure 1. To investigate the highly nonlinear impact of changes in peak wind speed on dust emission independently of changes in soil parameters, the recently established Dust Uplift Potential (*DUP*) diagnostic parameter [*Marsham et al*., 2011] is used: *DUP* = *U*^3^ (1 + *U*_t_/*U*) (1 – *U*_t_^2^/*U*^2^), where *U* is the measured wind speed and *U*_t_ is a threshold wind speed for dust emission. *DUP* uses the wind speed dependent components of a widely used dust uplift parameterization [*Marticorena and Bergametti*, 1995] but assumes a simple idealized soil with a single, constant threshold value *U*_t_. Trends and correlations with the North Atlantic Oscillation (NAO) were assessed using the Jones NAO index [*Jones et al*., 1997] (data available from http://www.cru.uea.ac.uk). Vegetation changes were assessed using the normalized difference vegetation index (NDVI) data [*Tucker et al*., 2005] from the Advanced Very High Resolution Radiometer remote sensing instrument. This widely used proxy for vegetation in the Sahel [*Huber and Fensholt*, 2011] is well suited for studies in semiarid areas [*Olsson et al*., 2005]. NDVI data, provided on an 8 km by 8 km grid, was obtained from the Global Inventory Modeling and Mapping Studies (GIMMS) database (http://www.landcover.org) for the time period 1984–2006.

[6] An analysis of the possibility of artificial trends due to instrument changes (as in *Klink* [1999]) suggests potential problems at Tombouctou (see auxiliary material). Ultimately, we decided to leave the station in. Removing it would actually strengthen the overall trends discussed in the paper.

## 3. Results

**Figure 2:**
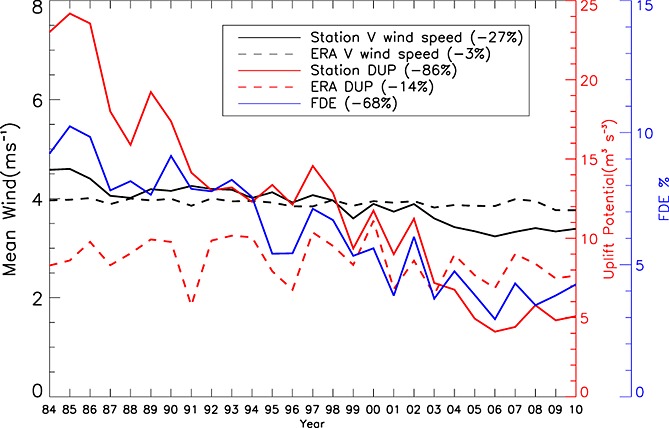
Trends in mean annual 10 m wind speed (*V*, black lines), dust uplift potential (*DUP*, red lines), and frequency of dust events (*FDE*, blue line) from observations averaged over seven surface stations in the Sahel (see Figure 1 for locations; solid lines) and ERA-Interim reanalysis averaged over the blue box shown in Figure 1 (dashed lines) for the time period 1984–2010. Numbers in brackets in the legend indicate the relative change over the time period estimated from the linear trend line as in Table 1. Definitions of *DUP* and *FDE* are given in section 2. Note that there is no *FDE* from reanalysis data. A fixed threshold of 7 ms^–1^ was used for the *DUP* computations.

[7] Figure 2 shows long-term trends in wind speed and dust averaged over the seven Sahelian stations. Annual mean wind speeds (*V*) decrease from 4.6 ms^–1^ in the mid-1980s to 3.3 ms^–1^ in recent years (−27%; solid black line in Figure 2), consistent with previous studies [*Mahowald et al*., 2007]. Year-to-year variability is low. Six of the seven stations show a negative trend varying from −19 to −64%; only winds at Tombouctou increase (Table 1). The seven station average, mean annual *FDE* decreases dramatically from more than 10% in 1985 to just under 3% by 2006 (blue line in Figure 2), consistent with trends in visibility [*Mahowald et al*., 2007] and dust over the downstream tropical Atlantic [*Evan and Mukhopadhyay*, 2010; *Foltz and McPhaden*, 2008]. Again, all stations except Tombouctou show large negative trends (Table 1). A time series of *DUP* using a constant emission threshold of 7 ms^–1^, as used in *Chomette et al*. [1999], shows a dramatic reduction by 86% over the 27 year study period (red line, Figure 2) and has interstation variability largely consistent with that for mean wind (Table 1). The highly significant interannual correlations between the independently estimated *FDE* and mean wind (0.92), and *FDE* and *DUP* (0.93) reflect the strong control of wind speed on the occurrence of dust emission. Surprisingly, corresponding trends in regionally averaged mean wind and *DUP* computed from ERA-Interim reanalysis are substantially smaller (dashed lines in Figure 2) reaching only −3% (0.25 ms^–1^) and −14%, respectively.

**Table 1:** Key Trends and Characteristics for Individual Sahelian Stations (Locations in Figure 1)^1^

		Agadez	Gouré	Niamey	Gao	Tomb.	Nema	Nouakchott
1	% change in V	***−20***	***−64***	***−21***	***−37***	***47***	***−50***	***−19***
2	% change in FDE	−22	***−71***	***−63***	***−84***	−17	***−60***	***−94***
3	% change in DUP	***−49***	***−121***	***−65***	***−100***	15	***−112***	***−95***
4	% change in NDVI (SON)	**+12**	***+25***	**+13**	***+43***	***+19***	**+16**	+2.7
5	Season of largest V change	***JJA***	***SON***	***DJF***	***MAM***	SON^*^	***DJF***	***DJF***
6	Season of largest FDE change	**JJA**	***SON***	SON	***SON***	JJA^*^	JJA	**DJF**
7	Season of largest DUP change	***JJA***	***SON***	**SON**	***SON***	JJA^*^	***SON***	***MAM***
8	Station location In/out of city	in	out	in	out	out	out	in
9	Number of wind speed obs	35991	30618	57476	42438	41623	29777	67582

a Relative changes (in %) in 10 m mean wind (*V*), frequency of dust events (*FDE*) and dust uplift potential (*DUP*) in rows 1–3 are computed for 1984–2010 based on the linear trend. NDVI changes are calculated for the time period 1984–2006. Definitions of *V*, *FDE*, and *DUP* are given in section 2. Note that for some stations the *DUP* changes are so dramatic that the linear trend line crosses the zero axis, resulting in relative changes of more than 100%. The seasons of largest changes in rows 5–7 are based on relative changes computed in the same way, but for the four standard seasons December–February (DJF), March–May (MAM), June–August (JJA), and September–November (SON). All changes in rows 5–7 are negative except for *V*, *FDE*, and *DUP* at Tombouctou marked with “^*^”. The classification of station location in or outside of the main urban area was carried out on the basis of google earth images. The latter is often the case when airports were built remote from the city centers. Row 9 gives the number of available reports of wind speed for the period 1984–2010, for each station. Statistical significance at the 95% and 99% levels (90% and 95% for row 4) are denoted in bold and in bold Italics, respectively.

[8] We have also analyzed the trends for: (a) day/night-time time data, (b) data at each major SYNOP hour (00, 06, 12, and 18 UTC), and (c) data in each season. In each case the results are robust (see auxiliary material), supporting the hypothesis that the observed trends are real and not an artefact of a change in sampling through the period.

## 4. Discussion

**Figure 3:**
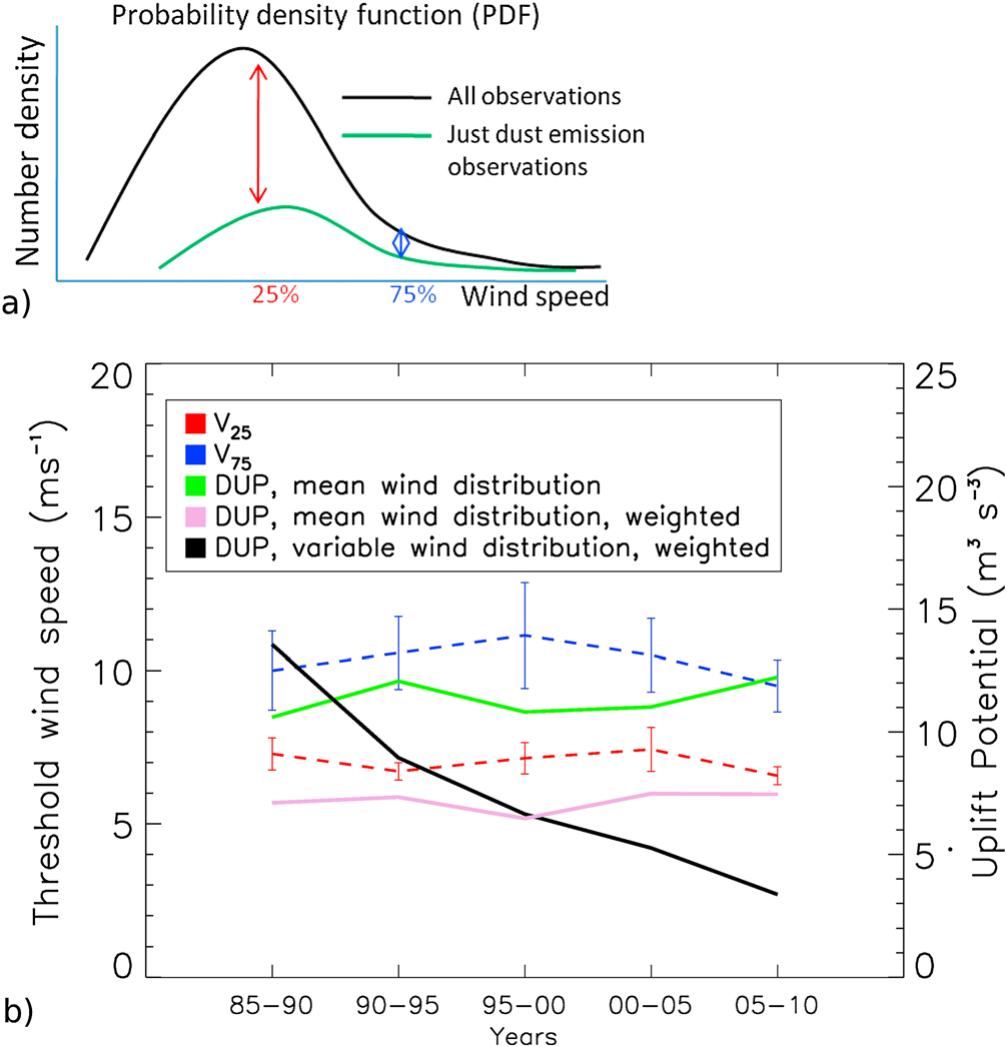
(a) Schematic illustrating the estimation of emission threshold wind velocities using probability density distributions for all wind observations and those during dust emission events. Probabilities of 25% (*v_25_*) and 75% (*v_75_*) were arbitrarily selected to characterize the range of wind speeds typical for the beginning of dust emission. (b) Time evolution of *v_25_* and *v_75_* threshold velocities computed for each station, for 5 year periods from 1985–2010, then averaged over all stations (dashed lines, left axis). Standard deviations of *v_25_* and *v_75_* wind speeds are given by the error bars. Corresponding *DUP* calculations are also shown (right axis) using (1) a mean wind distribution over the whole time period and the *v_25_* threshold velocity (green), (2) a mean wind distribution and a probability weighting (purple), and (3) a varying wind distribution representative of each 5 year period and a probability weighting (black).

[9] A change in emission thresholds is a possible contributor to the decrease in dust emission. Threshold values, which depend on soil and sediment characteristics [*Helgren and Prospero*, 1987], may have been affected by recent increases in rainfall [*Foltz and McPhaden*, 2008] and vegetation [*Fensholt et al*., 2012] over the Sahel. Calculating wind speed probability density functions of all observations and dust events separately allow for the determination of the wind speeds, *v25* and *v75*, at which the probability of dust emission is 25% and 75%, respectively (Figure 3a). *v25* and *v75* were computed at each station, for 5 year periods, from 1985 to 2010 and then averaged over the seven stations. *v25* varies around 7 ms^–1^ (the threshold assumed for the *DUP* computations in Figure 2); *v75* is 10 ms^–1^. Neither of the time series shows a clear trend, and temporal variations are not coherent (red and blue dashed lines in Figure 3b). Calculating *DUP* using the time-varying *v25* as the threshold and an identical mean wind distribution for each 5 year period shows relatively small variations (green line in Figure 3b). Repeating this calculation with a weighting that takes into account the changing probability for each 1 ms^–1^ wind speed bin, results in even less variability (purple line in Figure 3b). However, when the real wind distribution for each 5 year period is used, a sharp decrease in *DUP* is found (black line, Figure 3b), consistent with Figure 2 (solid red line). This suggests that changes in wind, and not emission thresholds, are the cause of the observed downward trends in dustiness.

[10] Another potential contributor is a change in the large-scale circulation over North Africa. Assuming that ERA-Interim is capable of representing such changes accurately, the small trend in ERA-Interim mean wind (Figure 2) suggests that, at least on an annual basis, such contributions are small. An inspection of individual seasons reveals that the largest relative trends of −7% and −31% (for *V* and *DUP*, respectively) are in boreal winter (DJF, Table S3). In this season the entire region is dominated by the northeasterly Harmattan winds (vectors in Figure 1) and the correlation between ERA-Interim and station *V* are at their seasonal peak of 0.71. Winter is also characterized by highly significant correlations of 0.77 between the NAO and *V*, and significant correlations of 0.58 between the NAO and *FDE* [*Engelstaedter et al*., 2006; *Hsu et al*., 2012]. A steady downward trend in the winter NAO Index since 1995 [*Osborn*, 2006] can explain the winter trend in ERA-Interim data and a part of the larger trend in station data.

[11] A factor that could explain both the downward trend in observed *V* and *DUP*, and the discrepancies with ERA-Interim, is an increase in surface roughness due to vegetation growth [*Roderick et al*., 2007; *Vautard et al*., 2010]. This would affect both station winds locally and the greater Sahel area, consistent with large-scale dust trends found in satellite data [*Evan and Mukhopadhyay*, 2010; *Foltz and McPhaden*, 2008]. Previous work using NDVI data found a substantial greening of the Sahel in recent decades [*Olsson et al*., 2005; *Fensholt et al*., 2012]. Given the short Sahelian rainy season from July–September, NDVI is likely to give the best results for the main growing season in late summer and autumn. For September–November (SON), six of the seven stations show a statistically significant positive trend in NDVI (Table 1), calculated from a 24 by 24 km^2^ area encompassing each station as in *Vautard et al*. [2010]. The large autumn trend is robust for the densely vegetated southern stations, while the two northernmost stations, Agadez and Nouakchott, have low mean values and weaker trends, which peak in winter (not shown). Vegetation is sparse in these locations and might not be captured very well by NDVI. Remarkably, the four stations with strong vegetation increases, Gouré, Niamey, Gao, and Nema, also have large negative trends in mean wind and *DUP*, particularly in autumn (Table 1), which support the link between high winds and vegetation. The largest *FDE* trends are split between autumn and the more active summer dust season, with the exception of Nouakchott (Table 1). For all stations combined however, autumn shows the largest relative changes for both *DUP* and *FDE* (Table S1). It should be pointed out that an increase in green vegetation is likely to affect roughness well beyond the main growing season [*Zender and Kwon*, 2005], which is not well represented in NDVI data designed for photosynthetically active plants.

[12] Vegetation can also contribute to the negative trends in winds through changes in the surface energy budget. Increased transpiration, plus possible larger evaporation from moister soils, will increase latent heat flux at the expense of sensible heating of the atmosphere. The reduction in daytime buoyancy of near-surface air tends to reduce turbulence and therefore gustiness. This effect should be well represented in station data but not in reanalyses, which capture only mean grid-scale winds. Globally, it has been suggested that roughness changes dominate over changes in daytime turbulence and evapotranspiration [*Vautard et al*., 2010]. Our data shows that absolute trends in mean wind and *DUP* averaged over the seven stations are more negative during the day than night (Table S5), which is consistent with this idea. Increased vegetation also reduces the area of bare soil available for dust emission. This may contribute to trends in dust, but cannot explain the corresponding trends in mean wind and *DUP*.

[13] An additional localized influence could come from a change in roughness and surface characteristics by man-made structures, as Sahelian cities have been growing substantially over the last decades [*Olsson et al*., 2005]. This would not be captured by reanalyses data at all. Past work using station data in China [*Guo et al*., 2011] and Australia [*McVicar et al*., 2008] found that urbanization can weaken the observed stilling effect relative to rural stations. Consistent with this, three of the four stations situated outside of the main urban areas, Gouré, Nema, and Gao, show steeper negative trends in both mean wind and *DUP*, with Tombouctou again being the exception (Table 1). Detailed modeling studies are needed to further corroborate and quantify this effect [*Guo et al*., 2011].

## 5. Conclusions

[14] The analysis presented here offers a new perspective on the recently observed dramatic decadal trends in dustiness over North Africa and the tropical North Atlantic. Our results suggest that these trends are related to a reduction in dust emission over the Sahel, associated with reduced peak winds rather than changes in emission threshold. Increased roughness and reduced turbulence, as a result of the observed “greening” in the Sahel, appears to be the main cause of weaker winds. Changes in the large-scale circulation, possibly associated with the downturn of the NAO, are secondary. The large discrepancy between station and reanalysis data demonstrates that a better representation of interannual to decadal roughness changes in global and regional models is urgently needed to improve the modeling and understanding of the global dust cycle. The results are consistent with strong downward trends in dustiness over the tropical North Atlantic (5°N–20°N), while no clear trends are found at higher latitudes (15°N–30°N), which are probably more strongly influenced by the very sparsely vegetated Sahara [*Evan and Mukhopadhyay*, 2010; *Chiapello et al*., 2005]. To quantify the highly disputed anthropogenic impacts [*Engelstaedter et al*., 2006] on dustiness, it is important to investigate to what extent agricultural activities in the Sahel could, or have, changed vegetation, roughness, wind, and ultimately dust emission.

## Key Points

- Decadal Sahel dust trends analyzed with surface observations and new diagnostics
- Wind-speed changes dominate over soil changes in recent dust emission decrease
- Vegetation-induced roughness changes are the main control on wind-speed trends

## References

[b1] Ackerman S, Cox S (1989). Surface weather observations of atmospheric dust over the southwest summer monsoon region. Meteorol. Atmos. Phys.

[b2] Brindley H, Knippertz P, Ryder C, Ashpole I (2012). A critical evaluation of the ability of the Spinning Enhanced Visible and Infrared Imager (SEVIRI) thermal infrared red-green- blue rendering to identify dust events: Theoretical analysis. J. Geophys. Res.

[b3] Chiapello I, Moulin C, Prospero JM (2005). Understanding the long-term variability of African dust transport across the Atlantic as recorded in both Barbados surface concentrations and large-scale Total Ozone Mapping Spectrometer (TOMS) optical thickness. J. Geophys. Res.

[b4] Chomette O, Legrand M, Marticorena B (1999). Determination of the wind-speed threshold for the emission of desert dust using satellite remote sensing in the thermal infrared. J. Geophys. Res.

[b5] Engelstaedter S, Washington R (2007). Atmospheric controls on the annual cycle of North African dust. J. Geophys. Res.

[b6] Engelstaedter S, Kohfeld KE, Tegen I, Harrison SP (2003). Controls of dust emissions by vegetation and topographic depressions: An evaluation using dust storm frequency data. Geophys. Res. Lett.

[b7] Engelstaedter S, Tegen I, Washington R (2006). North African dust emissions and transport. Earth Sci. Rev.

[b8] Evan AT, Mukhopadhyay S (2010). African dust over the northern tropical Atlantic: 1955–2008. J. Clim. Appl. Meteorol.

[b9] Evan AT, Heidinger AK, Knippertz P (2006). Analysis of winter dust activity off the coast of West Africa using a new 24-year over-water Advanced Very High Resolution Radiometer satellite dust climatology. J. Geophys. Res.

[b10] Fensholt R (2012). Greenness in semi-arid areas across the globe 1981–2007 - an Earth Observing Satellite based analysis of trends and drivers. Remote Sens. Environ.

[b11] Foltz GR, McPhaden MJ (2008). Trends in Saharan dust and tropical Atlantic climate during 1980–2006. Geophys. Res. Lett.

[b12] Goudie AS, Middleton NJ (2001). Saharan dust storms: nature and consequences. Earth Sci. Rev.

[b13] Guo H, Xu M, Hu Q (2011). Changes in near-surface wind speed in china: 1969–2005. Int. J. Climatol.

[b14] Helgren DM, Prospero JM (1987). Wind velocities associated with dust deflation events in the western Sahara. J. Clim. Appl. Meteorol.

[b15] Hsu NC, Gautam R, Sayer AM, Bettenhausen C, Li C, Jeong MJ, Tsay SC, Holben BN (2012). Global and regional trends of aerosol optical depth over land and ocean using SeaWiFS measurements from 1997 to 2010. Atmos. Chem. Phys.

[b16] Huber S, Fensholt R (2011). Analysis of teleconnections between AVHRR-based sea surface temperature and vegetation productivity in the semi-arid Sahel. Remote Sens. Environ.

[b17] Jones P, Jonsson T, Wheeler D (1997). Extension to the North Atlantic Oscillation using early instrumental pressure observations from Gibraltar and south-west Iceland. Int. J. Climatol.

[b18] Klink K (1999). Trends in mean monthly maximum and minimum surface wind-speeds in the conterminous United States, 1961 to 1990. Clim. Res.

[b19] Klose M, Shao Y, Karremann MK, Fink AH (2010). Sahel dust zone and synoptic background. Geophys. Res. Lett.

[b20] Knippertz P, Todd MC (2012). Mineral dust aerosols over the Sahara: Meteorological controls on emission and transport and implications for modeling. Rev. Geophys.

[b21] Mahowald NM, Ballantine JA, Feddema J, Ramankutty N (2007). Global trends in visibility: implications for dust sources. Atmos. Chem. Phys.

[b22] Marsham JH, Knippertz P, Dixon NS, Parker DJ, Lister GMS (2011). The importance of the representation of deep convection for modeled dust-generating winds over West Africa during summer. Geophys. Res. Lett.

[b23] Marticorena B, Bergametti G (1995). Modeling the atmospheric dust cycle: 1. Design of a soil derived dust emission scheme. J. Geophys. Res.

[b24] Mbourou G, Bertrand J, Nicholson S (1997). The diurnal and seasonal cycles of wind-borne dust over Africa north of the equator. J. Appl. Meteorol.

[b25] McVicar TR, Van Niel TG, Li LT, Roderick ML, Rayner DP, Ricciardulli L, Donohue RJ (2008). Wind speed climatology and trends for Australia, 1975–2006: Capturing the stilling phenomenon and comparison with near-surface reanalysis output. Geophys. Res. Lett.

[b26] Moulin C, Chiapello I (2004). Evidence of the control of summer atmospheric transport of African dust over the Atlantic by Sahel sources from TOMS satellites (1979–2000). Geophys. Res. Lett.

[b27] Olsson L, Eklundh L, Ardo J (2005). A recent greening of the Sahel – trends, patterns and potential causes. J. Arid Environ.

[b28] Osborn TJ (2006). Recent variations in the winter North Atlantic oscillation. Weather.

[b29] Prospero JM, Lamb PJ (2003). African droughts and dust transport to the Caribbean: Climate change implications. Science.

[b30] Roderick ML, Rotstayn LD, Farquhar GD, Hobbins MT (2007). On the attribution of changing pan evaporation. Geophys. Res. Lett.

[b31] Tucker C, Pinzon J, Brown M, Slayback D, Pak E, Mahoney R, Vermote E, El Saleous N (2005). An extended AVHRR 8-km NDVI dataset compatible with MODIS and spot vegetation NDVI data. Int. J. Remote Sens.

[b32] Vautard R, Cattiaux J, Yiou P, Thepaut J-N, Ciais P (2010). Northern hemisphere atmospheric stilling partly attributed to an increase in surface roughness. Nat. Geosci.

[b33] Zender C, Kwon E (2005). Regional contrasts in dust emission responses to climate. J. Geophys. Res.

