# Supplementary figures and images for: Are vegetation-related roughness changes the cause of the recent decrease in dust emission from the Sahel?
Source: Geophys Res Lett. 2013 May 13;40(9):1868–72. doi: 10.1002/grl.50273 (PMC4373181; doi:10.1002/grl.50273)

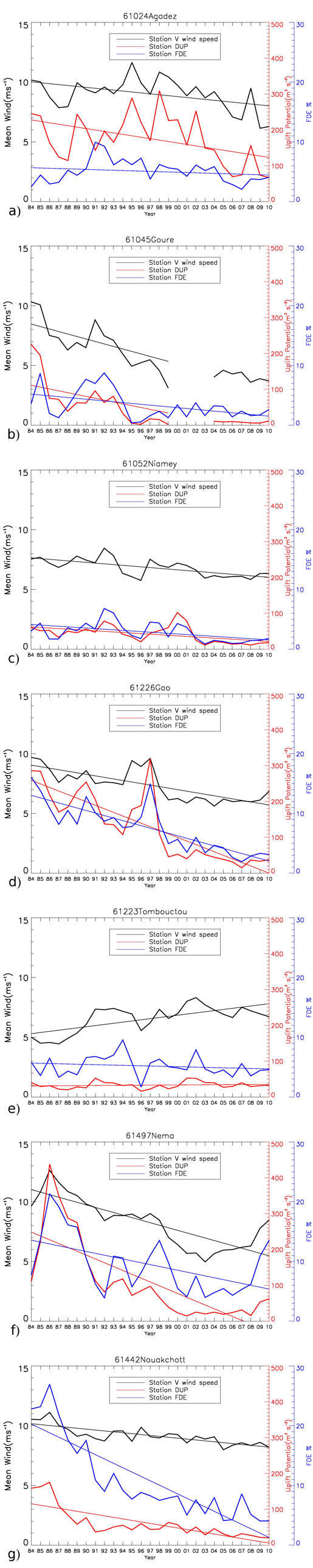

Supplement: Supplementary file 1 [file grl0040-1868-sd1.jpg]

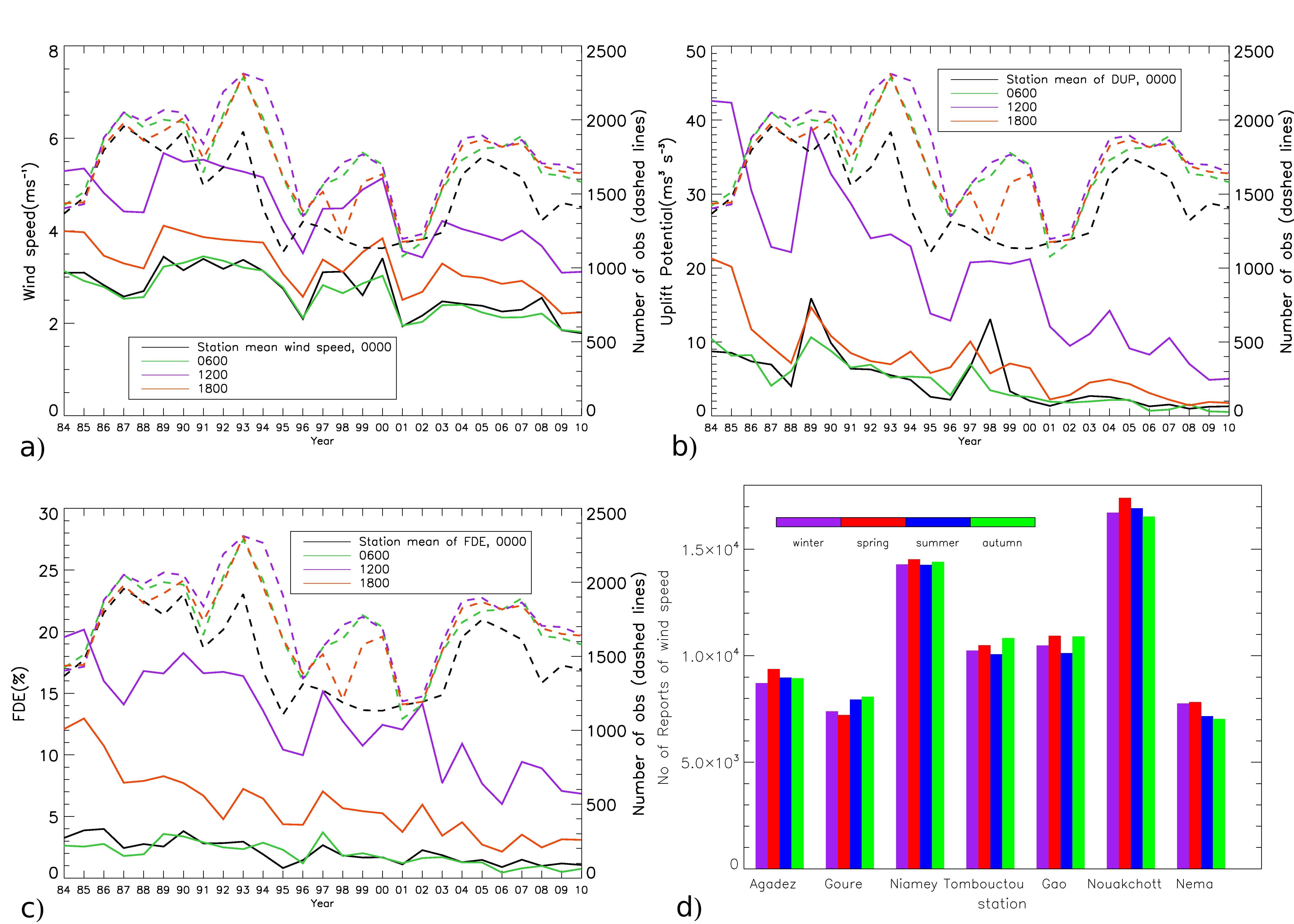

Supplement: Supplementary file 2 [file grl0040-1868-sd2.jpg]

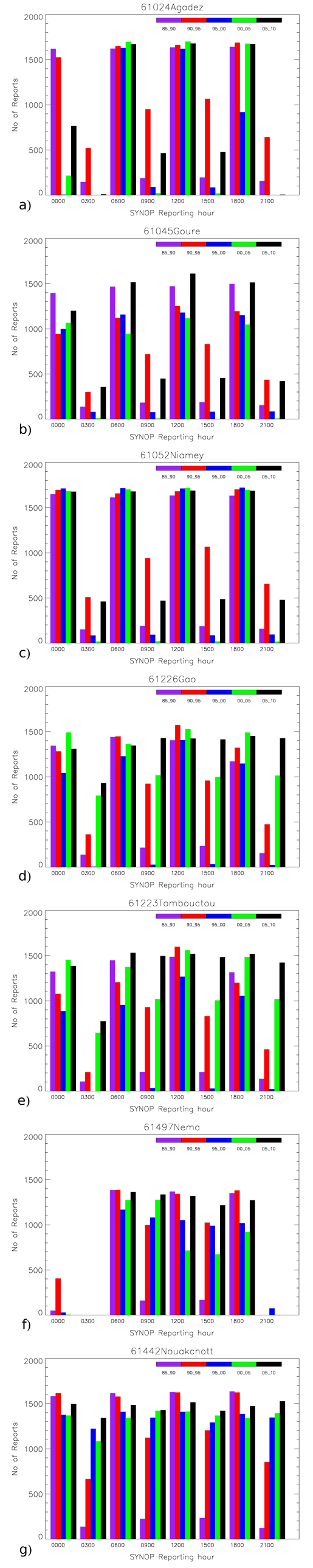

Supplement: Supplementary file 3 [file grl0040-1868-sd3.jpg]

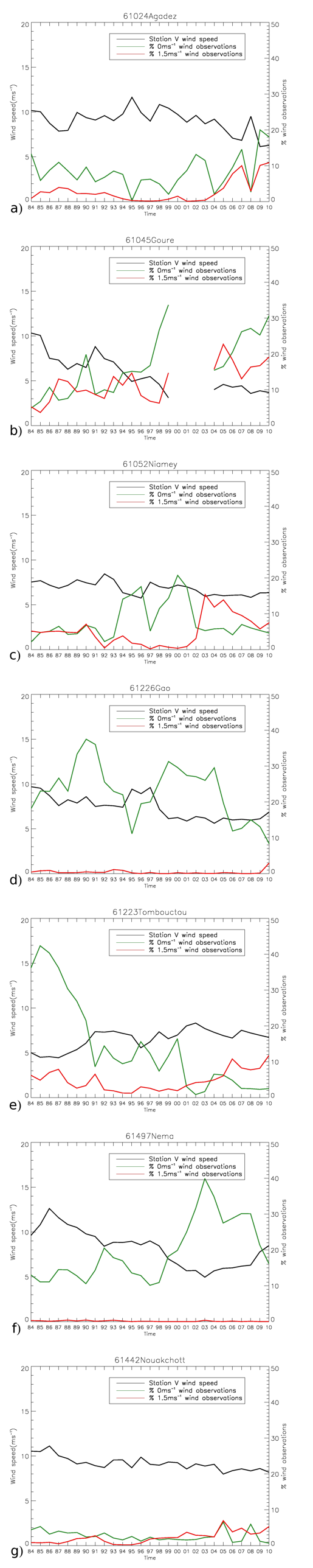

Supplement: Supplementary file 4 [file grl0040-1868-sd4.jpg]

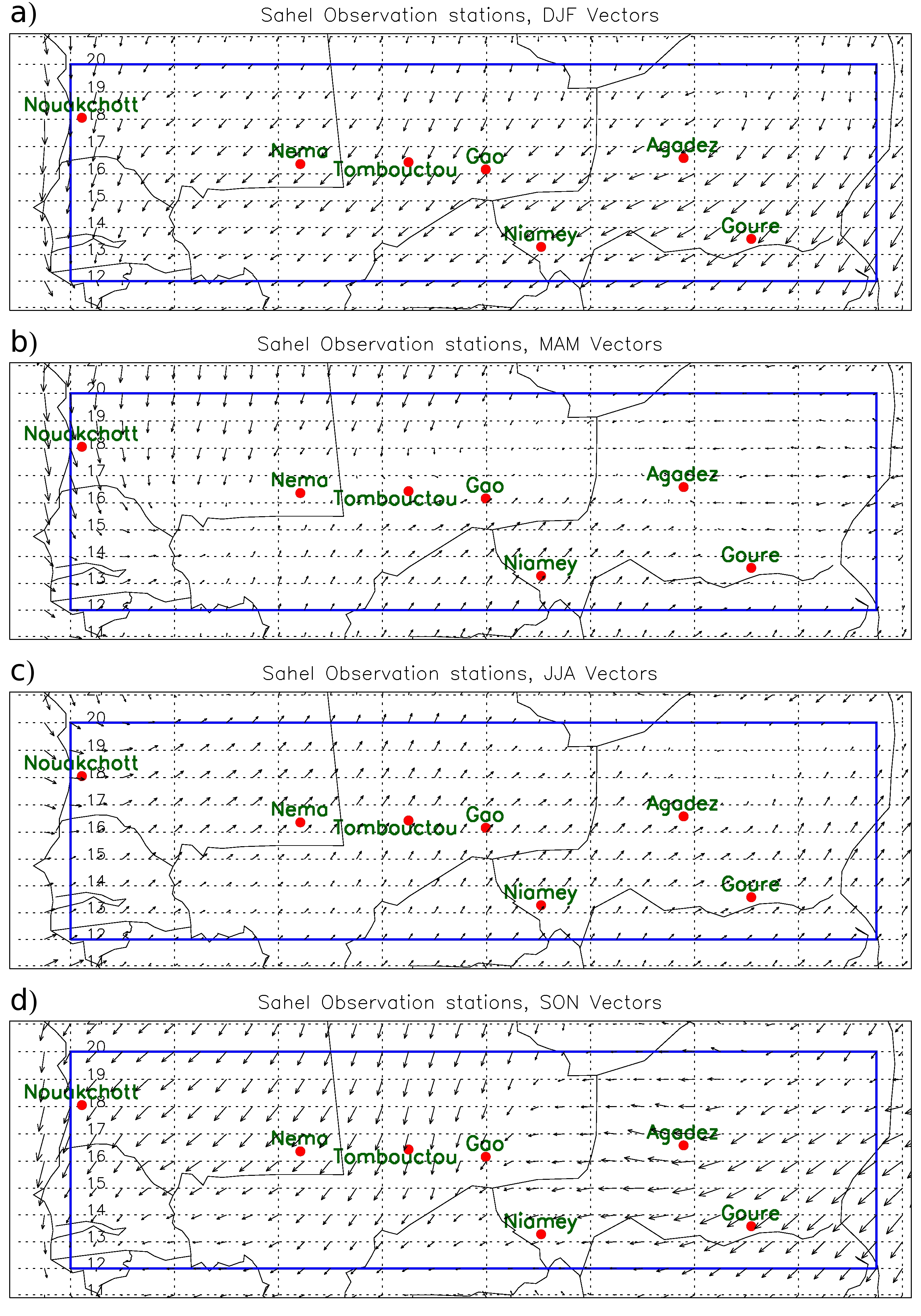

Supplement: Supplementary file 5 [file grl0040-1868-sd5.jpg]
