# Supplementary material for: Are vegetation-related roughness changes the cause of the recent decrease in dust emission from the Sahel?
Source: Geophys Res Lett. 2013 May 13;40(9):1868–72. doi: 10.1002/grl.50273 (PMC4373181; doi:10.1002/grl.50273)
Supplement: Supplementary file 6 [file grl0040-1868-sd6.doc]

**Tables**

**Auxiliary Table 1: Seasonality of wind speed and dust trends.**

|  |  | **DJF** | **MAM** | **JJA** | **SON** | **Year** |
| --- | --- | --- | --- | --- | --- | --- |
| **1** | **% change in V** | ***-31*** | ***-23*** | ***-26*** | ***-30*** | ***-27*** |
| **2** | **% change in FDE** | ***-49*** | **-34** | ***-47*** | ***-72*** | ***-68*** |
| **3** | **% change in DUP** | ***-84*** | ***-83*** | ***-90*** | ***-91*** | ***-86*** |
| **4** | **% change in V > 5 m s^–1^** | ***-16*** | ***-14*** | ***-21*** | ***-30*** | ***-20*** |
| **5** | **% change in V < 5 m s^–1^** | **-4** | **6** | **4** | **-2** | **1** |
| **6** | **V / FDE corr** | ***0.74*** | **0.55** | **0.41** | ***0.83*** | ***0.92*** |
| **7** | **V / DUP corr** | ***0.92*** | ***0.93*** | ***0.88*** | ***0.76*** | ***0.95*** |
| **8** | **FDE / DUP corr** | ***0.61*** | **0.53** | **0.46** | ***0.72*** | ***0.93*** |

**Auxiliary Table 1:** Relative changes (in %) for rows 1–5 are computed in the same way as rows 1–3 in Table 1 in the main text. Rows 6–8 give linear correlation coefficients for seven-station means of V, FDE, and DUP. Statistical significance of trends and correlations at the 95% and 99% levels are denoted in bold and in bold italics, respectively.

**Auxiliary Table 2: Instrument degradation trends and correlations**

|  |  | **Agadez** | **Gouré** | **Niamey** | **Gao** | **Tomb.** | **Nema** | **Nouakchott** |
| --- | --- | --- | --- | --- | --- | --- | --- | --- |
| **1** | **0 ms^-1^ trend** | **-52** | ***-186*** | **-33** | ***39*** | ***112*** | ***-190*** | **49** |
| **2** | **<1.5 ms^-1^ trend** | ***-1228*** | ***-176*** | **-220** | **-2** | **-119** | ***99*** | ***-1909*** |
| **3** | **Correlation:**  **<1.5 ms^-1^/0 ms^-1^** | ***0.65*** | **0.52** | **-0.4** | **-0.29** | **-0.07** | **-0.16** | **-0.02** |

**Auxiliary Table 2.** Rows 1 and 2 contain the 0 ms^-1^ trend (green lines) and <1.5 ms^-1^ trend (red lines) values as plotted in Aux. Figure 3. Row 3 gives the corresponding linear correlation. Statistical significance of trends and correlations at the 95% and 99% levels are denoted in bold and in bold italics, respectively.

**Auxiliary Table 3: Seasonality of ERA mean wind and DUP**

|  |  | **DJF** | **MAM** | **JJA** | **SON** | **Year** |
| --- | --- | --- | --- | --- | --- | --- |
| **1** | **% change in ERA V** | **-7** | **-4** | **2** | ***-5*** | ***-3*** |
| **2** | **% change in ERA DUP** | **-31** | **-5** | **2** | **-27** | **14** |
| **3** | **ERA V / ERA DUP correlation** | ***0.85*** | ***0.83*** | **0.50** | ***0.80*** | ***0.60*** |
| **4** | **ERA V / Obs V correlations** | ***0.71*** | **0.56** | **-0.07** | **0.53** | **0.56** |

**Auxiliary Table 3:** Seasonality of ERA mean wind and DUP. Relative changes (in %) of ERA-Interim mean wind V and DUP. Rows 1 and 2 are computed in the same way as rows 1–3 in Table 1 in the main text. Statistical significance of trends and correlations at the 95% and 99% levels are denoted in bold and in bold italics, respectively.

**Auxiliary Table 4: Correlations of mean wind and observed dust with the NAO index.**

|  |  | **DJF** | **MAM** | **JJA** | **SON** | **Year** |
| --- | --- | --- | --- | --- | --- | --- |
| **1** | **V ERA** | ***0.77*** | **0.31** | **-0.21** | **0.43** | **0.58** |
| **2** | **V OBS** | **0.5** | **0.14** | **0.43** | **0.28** | **0.46** |
| **3** | **FDE** | **0.58** | **0.36** | **0.26** | **0.3** | **0.52** |

**Auxiliary Table 4**: Correlations of mean wind and observed dust with the NAO index. Seasonal correlations of ERA-Interim mean wind V (row 1), observation mean wind V (row 2), and observed FDE (row 3) with the seasonal Jones NAO Index (as described in Section 2). Significance of trends and correlations at the 95% and 99% levels are denoted in bold and in bold italics, respectively.

**Auxiliary Table 5: Day/night station trends comparison**

|  |  |  | **DJF** | **MAM** | **JJA** | **SON** | **Year** |
| --- | --- | --- | --- | --- | --- | --- | --- |
| **Absolute trends (ms^-1^ a^-1^)** | **1** | **Day V** | ***-0.07*** | ***-0.05*** | ***-0.05*** | ***-0.06*** | ***-0.06*** |
|  | **2** | **Night V** | ***-0.09*** | ***-0.05*** | ***-0.05*** | ***-0.04*** | ***-0.04*** |
|  | **3** | **Day DUP** | ***-9.5*** | ***-8.1*** | ***-5.5*** | ***-4.9*** | ***-7*** |
|  | **4** | **Night DUP** | ***-3.8*** | **-2.6** | ***-3.8*** | ***-1.7*** | ***-3*** |
| **relative change (%)** | **5** | **Day V** | ***-30*** | ***-25*** | ***-27*** | ***-36*** | ***-28*** |
|  | **6** | **Night V** | **-59** | ***-35*** | ***-39*** | ***-53*** | ***-31*** |
|  | **7** | **Day DUP** | ***-83*** | ***-86*** | ***-87*** | ***-92*** | ***-86*** |
|  | **8** | **Night DUP** | ***-105*** | ***-91*** | ***-98*** | ***-79*** | ***-97*** |

**Auxiliary Table 5:** Day/night station trends comparison. Seasonal absolute and relative trends of day and night data from station averaged observations. Absolute trends in rows 1–4 are calculated as the average change in wind speed per year, while rows 5–8 represent the total change in wind speed during the study period 1984–2010 as a % of the initial value. Significance of trends at the 95% and 99% levels are denoted in bold and in bold italics, respectively.
