# Supplementary material for: Are vegetation-related roughness changes the cause of the recent decrease in dust emission from the Sahel?
Source: Geophys Res Lett. 2013 May 13;40(9):1868–72. doi: 10.1002/grl.50273 (PMC4373181; doi:10.1002/grl.50273)
Supplement: Supplementary file 8 [file grl0040-1868-sd8.pdf]

Auxiliary Material

Trend Analysis

Auxiliary Figure 1 shows time series and trend lines in analogy to Figure 2 of the main paper, but for the seven individual stations. This helps to illustrate station-to-station variations in addition to Table 1 of the main paper. Auxiliary Table 1 adds further detail to Table 1 of the main paper by providing trends and correlations between wind and dust variables for individual seasons.

Data Availability

To check that the station trends are not the symptom of biases in data availability we computed trends at the main SYNOP hours of 0000, 0600, 1200 and 1800 UTC for mean wind, DUP, and FDE. Aux Figs.. 2a,b and c show consistently negative trends through the day and night for all parameters. There are no correlations above 0.5 between the wind parameters and the number of observations. Although the number of available observations at each station varies from 67582 at Nouakchott to 29777 at Nema (Table 1), there are no notable seasonal differences in the number of available reports (Fig. 2d), which increases confidence in the negative seasonal trends we observe (Table 1, rows 5-7). Generally, the number of reports at the hours 0300, 0900, 1500 and 2100 UTC have increased, but these make a relatively small contribution to the dataset anyway (Aux Fig 3).

Instrument Issues

As SYNOP observations are reported in knots, we adjust our analysis to compare the number of reports of 0 kts with reports of 1, 2, and 3 kts. In Auxiliary Fig. ~~2~~4 we refer to the two different series as 0 ms^{-1} and 1.5 ms^{-1}, as 3 kts is equal to 1.54 ms^{-1}. We calculate the

significance of trends in 1.5 ms^{-1} and 0 ms^{-1} reports as well as the correlations between mean wind and 0 ms^{-1} reports and 0 ms^{-1} and 1.5 ms^{-1} (Aux Table 2). Agadez, Nema, Gao, and Nouakchott do not show any suspicious behavior. A significant negative correlation of -0.4 between 0 ms^{-1} and 1.5 ms^{-1} is found at Niamey (Aux Fig. ~~2c~~4c, Aux Table 2). This appears to be mainly due to a period from the mid-1990s to the early 2000s when the number of 0 ms^{-1} reports is significantly enhanced (Aux Fig. ~~2c~~4c). The reason for this is not clear, but the good correspondence between the mean wind V, DUP, and the independently measured FDE shown in Aux Fig. 1c suggests that there is no significant influence on our analysis. Gouré has no available wind data in the period 2000 to 2003 (Aux Fig. ~~2b~~4b). The high percentage of 0 ms^{-1} reports just before this period signify a problem with the instrument, which might have been replaced after the gap. The continuation of the trend after the gap, and good correspondence with the independently measured FDE, support the usefulness of the record from this station. The steep drop in the percentage of 0 ms^{-1} reports from 1985 to 1991 in the Tombouctou time series (Aux Fig. ~~2e~~4e) is suspicious. However, for 1984–1992 alone, when 0 ms^{-1} reports are high, the correlation with 1.5 ms^{-1} is insignificant. The large percentage of 0 ms^{-1} reports at the start of the record is reflected in the mean wind and therefore contributes to the overall positive mean wind trend, which is opposite of what is observed at the other stations (Table 1.). For the remaining period (1993–2010) the correlation between 0 ms^{-1} and 1.5 ms^{-1} is highly significant at -0.64. This would be consistent with instrument degradation, but the negative trend in the number of 0 ms^{-1} reports does not support this (Aux Fig. ~~2e~~4e, Aux table 2). We chose to include Tombouctou because ultimately, this analysis did not produce any clear signs of instrument degradation.

ERA-Interim versus station observations

In the main paper, various parameters averaged over the seven Sahelian stations are compared to ERA-Interim re-analysis averaged over a larger box encompassing these stations (blue box in Aux. Figure ~~4~~5). It is ~~an~~ interesting and valid to question ~~to~~ what extent the stations can be regarded representative for this larger area. The seasonal cycle of 10-m winds shows how the Sahel changes from predominantly northeasterly Harmattan winds in DJF and SON to the southwesterly monsoon winds in JJA (Aux. Fig. ~~3~~5). In these seasons, the wind field is quite homogeneous over the entire ERA-Interim box and can therefore be compared to the station mean. The only critical season is MAM (Aux Fig. ~~3b~~5b) when the region is in transition from Harmattan to monsoon flow such that the stations might not necessarily fully represent the regime over the entire box well. In addition, station data will be affected by the local environment surrounding it in any given season, leading to disagreement with ERA data. This is particularly pronounced in summer, when deep moist convection creates dramatic changes in wind on small time and space scales, which are most likely not well represented in ERA in general, leading to low correlations between stations and ERA on an ~~interannual~~inter-annual basis (Aux. Table 3, row 4). Correlations in all other seasons are above 0.5 peaking in DJF with 0.71. This is also the time when ERA mean winds are correlated highest with ERA DUP (Aux. Table 3, row 3) and when largest trends in ERA data are observed (Aux. Table 3, rows 1–2). Not surprisingly, winter is also the season when correlations with the NAO are highest. This holds for ERA winds and station-observed winds and FDE (Aux. Table 4).

Changes in roughness versus surface heat exchange

A change in vegetation cover will affect both roughness and the exchange of sensible and latent heat between the surface and the atmosphere via evapo-transpiration. More latent

heating implies less sensible heating and therefore less turbulence and weaker winds. The latter can be expected to be only significant during the day and during the moist part of the year, i.e., JJA and SON. The fact that station-mean trends in wind and DUP are negative day and night throughout the year (Aux. Table 5) indicates very strongly that the roughness effect dominates. Absolute changes in mean wind and DUP are greater during the day, when mean values are larger. Relative changes are slightly greater at night, except for DUP during the vegetation maximum in SON (Aux. Table 5, rows 7 and 8). This suggests that the influence of latent heating may be a little more pronounced in this season.

Reference

Klink, K. (1999), Trends in mean monthly maximum and minimum surface wind-speeds in the conterminous United States, 1961 to 1990. *Clim. Res.*, *13*, 193–205, doi:10.3354/cr013193.

Auxiliary Figures & Tables

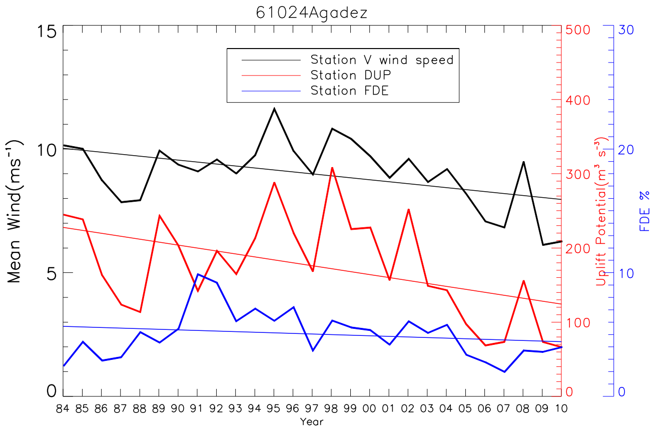

a)

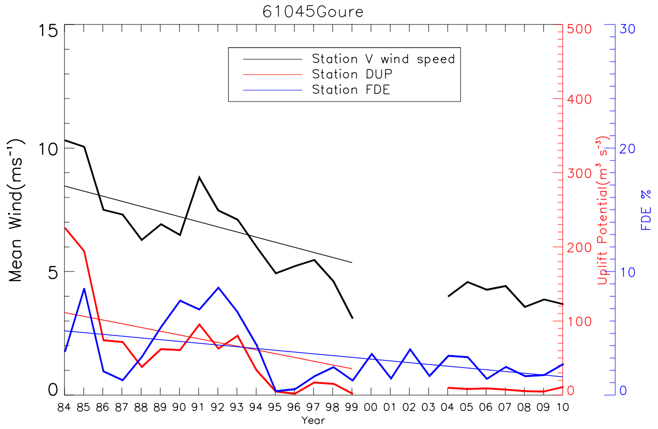
61045Goure
Station V wind speed
Station DUP
Station FDE
Mean Wind(ms-1)
Uplift Potential(m3 s-3)
FDE %
Year

87 **b)**

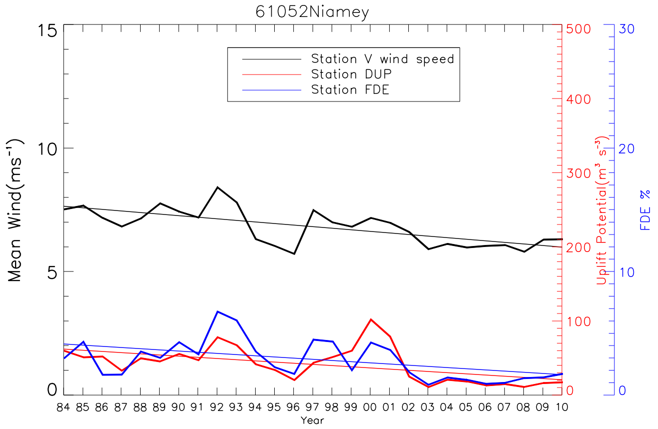
61052Niamey
Station V wind speed
Station DUP
Station FDE
Mean Wind(ms-1)
Uplift Potential(m3 s-3)
FDE %
Year

88 **c)**

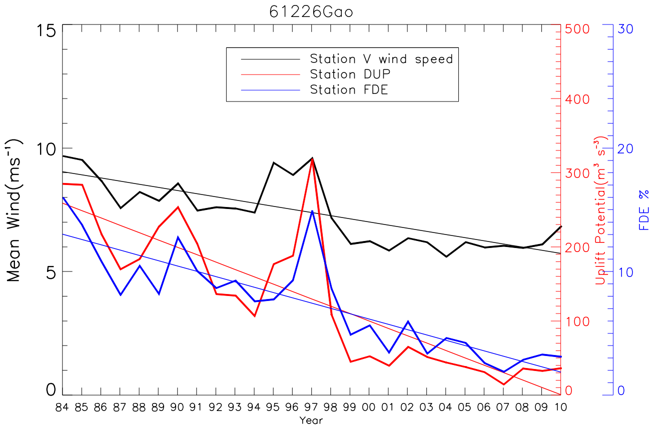
61226Gao
Station V wind speed
Station DUP
Station FDE
Mean Wind(ms^{-1})
Uplift Potential(m^3 s^{-3})
FDE %
Year

d)

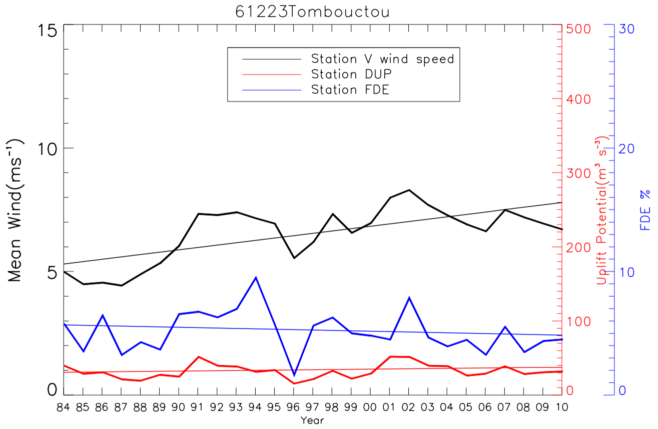
61223Tombouctou
Station V wind speed
Station DUP
Station FDE
Mean Wind(ms^{-1})
Uplift Potential(m^3 s^{-3})
FDE %
Year

e)

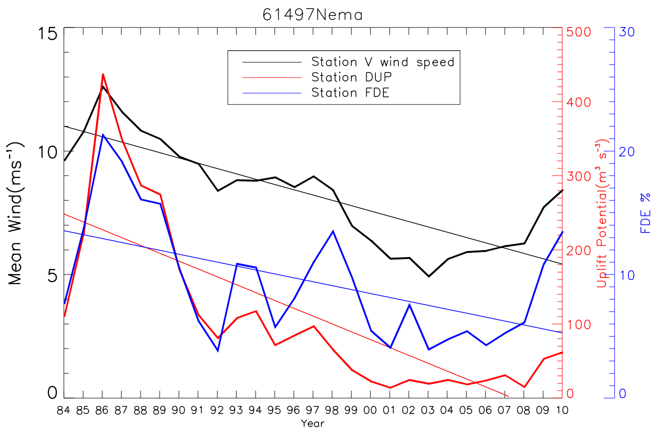

f)

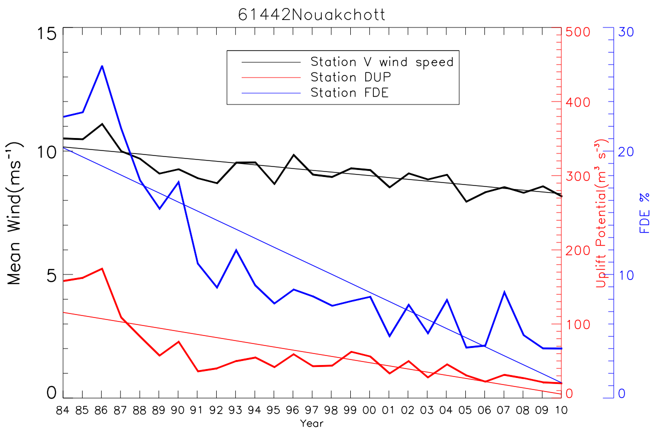

g)

Formatted: Font: Not Italic

Auxiliary Figure 1: Time series and trends separated by station. Same as Figure 2 in the main paper, with trends in mean wind V, DUP, and FDE given in black, red, and blue but for the individual stations of a) Agadez, b) Gouré, c) Niamey, d) Gao, e) Tombouctou, f) Nema, and g) Nouakchott. Trend values for each station are given in Table 1.

Formatted: Font: Not Bold

Formatted: Centered

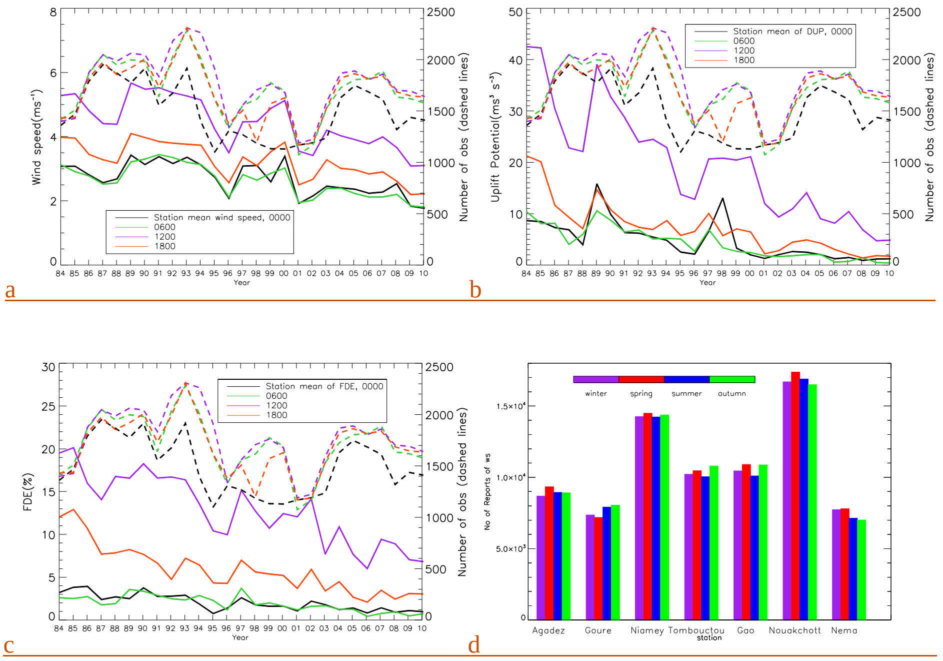

Auxiliary Figure 2. a) Trends in mean wind, averaged over the seven stations for the main SYNOP hours 0000 (black), 0600 (green), 1200 (purple) and 1800 UTC (orange). Number of wind speed observations are given by the same colors for the same hours with the dashed lines. b) same as a) but for DUP. c) same as a) but for FDE. d) Number of wind speed observations reported each season for each of the seven stations.

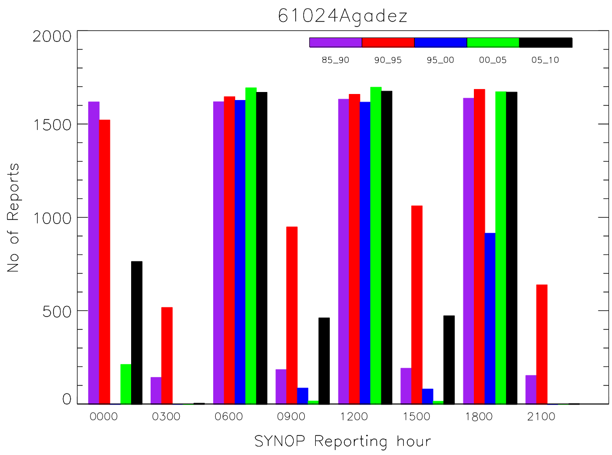

61024Agadez
85_90
90_95
95_00
00_05
05_10
2000
1500
1000
500
0
No of Reports
0000
0300
0600
0900
1200
1500
1800
2100
SYNOP Reporting hour

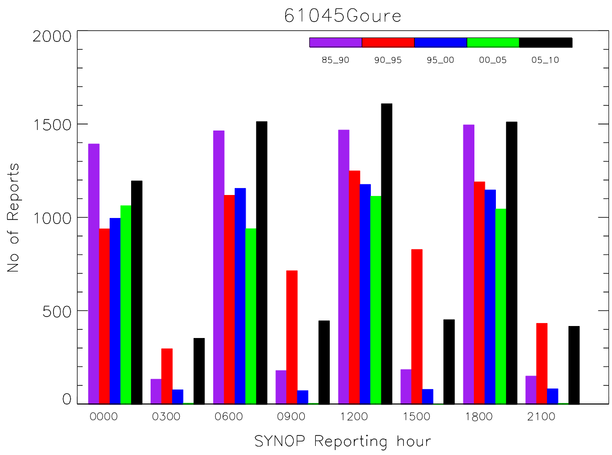
61045Goure
85_90
90_95
95_00
00_05
05_10
2000
1500
1000
500
0
No of Reports
0000
0300
0600
0900
1200
1500
1800
2100
SYNOP Reporting hour

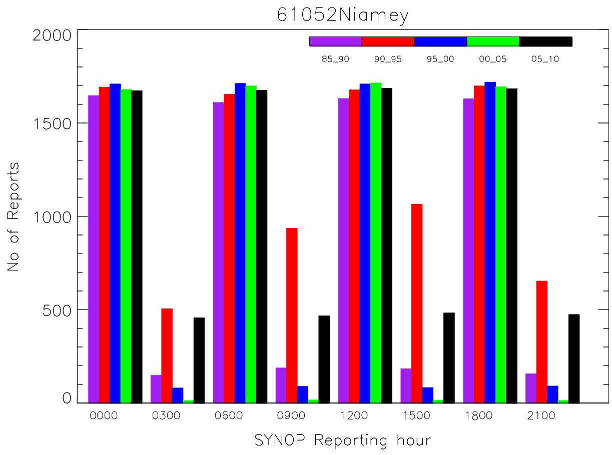
61052Niamey
85_90
90_95
95_00
00_05
05_10
2000
1500
1000
500
0
No of Reports
0000
0300
0600
0900
1200
1500
1800
2100
SYNOP Reporting hour

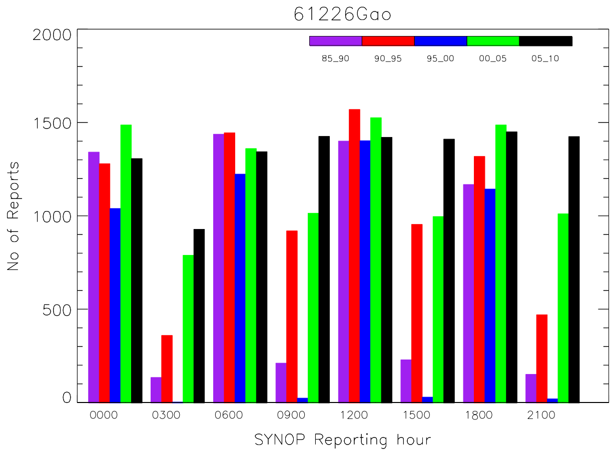
61226Gao
85_90
90_95
95_00
00_05
05_10
2000
1500
1000
500
0
No of Reports
0000
0300
0600
0900
1200
1500
1800
2100
SYNOP Reporting hour

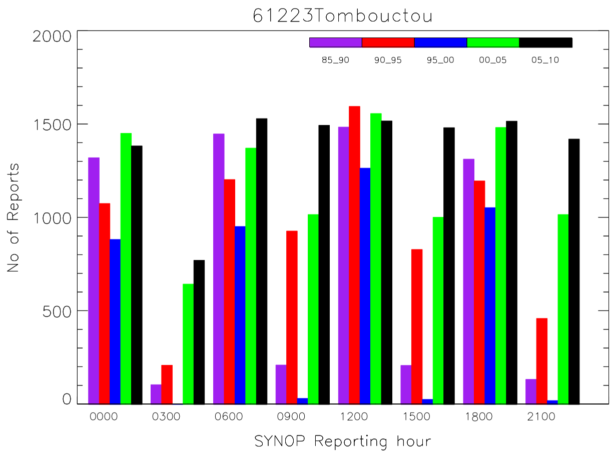
61223Tombouctou
85_90
90_95
95_00
00_05
05_10
No of Reports
0
500
1000
1500
2000
0000
0300
0600
0900
1200
1500
1800
2100
SYNOP Reporting hour

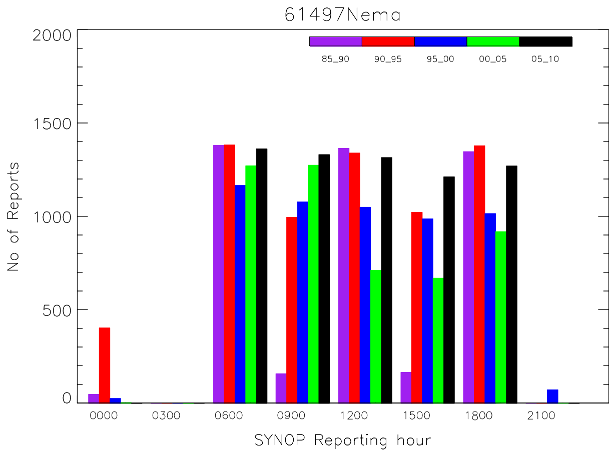
61497Nema
85_90
90_95
95_00
00_05
05_10
No of Reports
0
500
1000
1500
2000
0000
0300
0600
0900
1200
1500
1800
2100
SYNOP Reporting hour

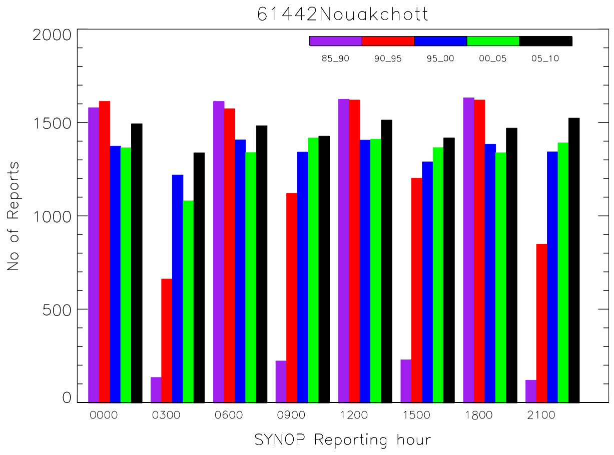
61442Nouakchott
85_90
90_95
95_00
00_05
05_10
No of Reports
0
500
1000
1500
2000
0000
0300
0600
0900
1200
1500
1800
2100
SYNOP Reporting hour

Auxiliary Figure 3: Bar plots of the number of observations at each SYNOP hour, for each 5-year period between 1985 and 2010 at: a) Agadez, b) Gouré, c) Niamey, d) Gao, e) Tombouctou, f) Nema, and g) Nouakchott.

Auxiliary Table 1: Seasonality of wind speed and dust trends.

		**DJF**	**MAM**	**JJA**	**SON**	**Year**
**1**	**% change in V**	***-31***	***-23***	***-26***	***-30***	***-27***
**2**	**% change in FDE**	***-49***	**-34**	***-47***	***-72***	***-68***
**3**	**% change in DUP**	***-84***	***-83***	***-90***	***-91***	***-86***
**4**	**% change in V > 5 m s^{-1}**	***-16***	***-14***	***-21***	***-30***	***-20***
**5**	**% change in V < 5 m s^{-1}**	**-4**	**6**	**4**	**-2**	**1**
**6**	**V / FDE corr**	***0.74***	**0.55**	**0.41**	***0.83***	***0.92***
**7**	**V / DUP corr**	***0.92***	***0.93***	***0.88***	***0.76***	***0.95***
**8**	**FDE / DUP corr**	***0.61***	**0.53**	**0.46**	***0.72***	***0.93***

Auxiliary Table 1: Relative changes (in %) for rows 1–5 are computed in the same way as rows 1–3 in Table 1 in the main text. Rows 6–8 give linear correlation coefficients for seven-station means of V, FDE, and DUP. Statistical significance of trends and correlations at the 95% and 99% levels are denoted in bold and in bold italics, respectively.

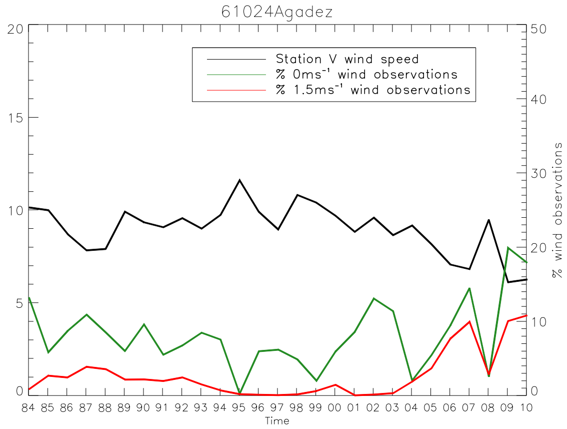
61024Agadez
Station V wind speed
% 0ms⁻¹ wind observations
% 1.5ms⁻¹ wind observations
% wind observations
Time
84 85 86 87 88 89 90 91 92 93 94 95 96 97 98 99 00 01 02 03 04 05 06 07 08 09 10
0 5 10 15 20
0 10 20 30 40 50

a)

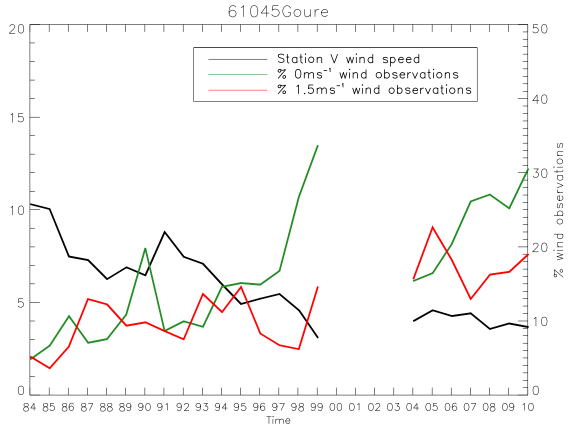
61045Goure
Station V wind speed
% 0ms⁻¹ wind observations
% 1.5ms⁻¹ wind observations
% wind observations
Time
84 85 86 87 88 89 90 91 92 93 94 95 96 97 98 99 00 01 02 03 04 05 06 07 08 09 10
0 5 10 15 20
0 10 20 30 40 50

b)

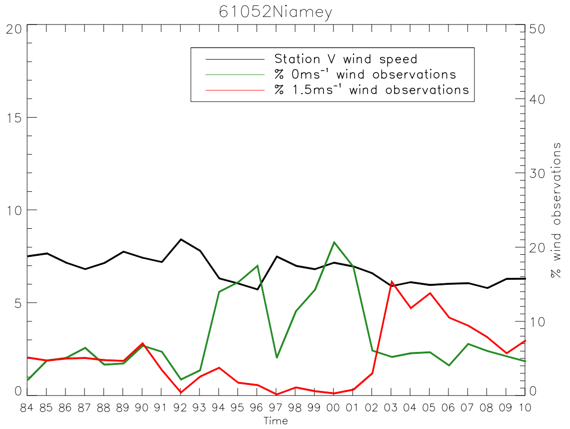
61052Niamey
Station V wind speed
% 0ms⁻¹ wind observations
% 1.5ms⁻¹ wind observations
% wind observations
Time
84 85 86 87 88 89 90 91 92 93 94 95 96 97 98 99 00 01 02 03 04 05 06 07 08 09 10

c)

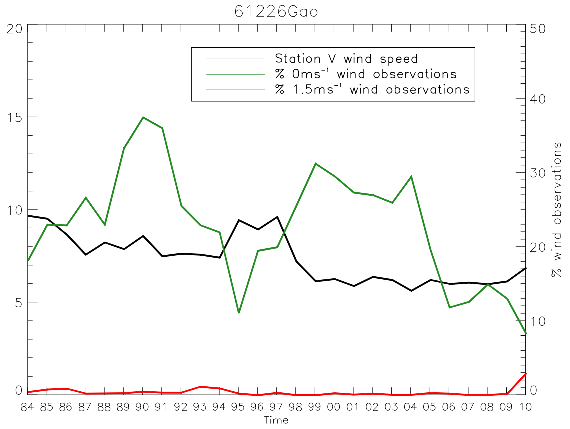
61226Gao
Station V wind speed
% 0ms⁻¹ wind observations
% 1.5ms⁻¹ wind observations
% wind observations
Time
84 85 86 87 88 89 90 91 92 93 94 95 96 97 98 99 00 01 02 03 04 05 06 07 08 09 10

d)

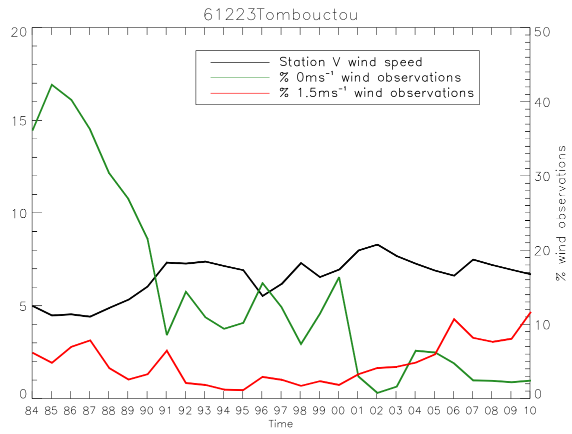
61223Tombouctou
Station V wind speed
% 0ms⁻¹ wind observations
% 1.5ms⁻¹ wind observations
% wind observations
Time
84 85 86 87 88 89 90 91 92 93 94 95 96 97 98 99 00 01 02 03 04 05 06 07 08 09 10
0 5 10 15 20
0 10 20 30 40 50

e)

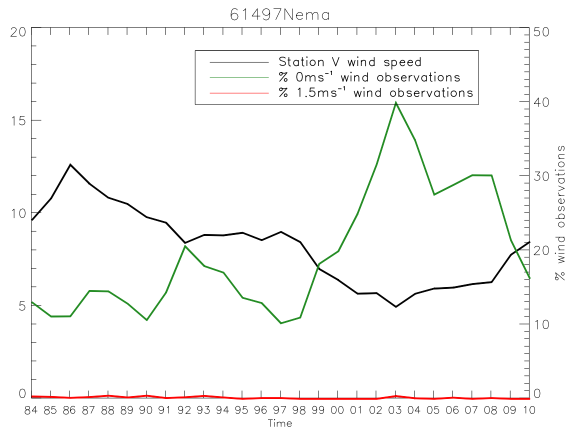
61497Nema
Station V wind speed
% 0ms⁻¹ wind observations
% 1.5ms⁻¹ wind observations
% wind observations
Time
84 85 86 87 88 89 90 91 92 93 94 95 96 97 98 99 00 01 02 03 04 05 06 07 08 09 10
0 5 10 15 20
0 10 20 30 40 50

f)

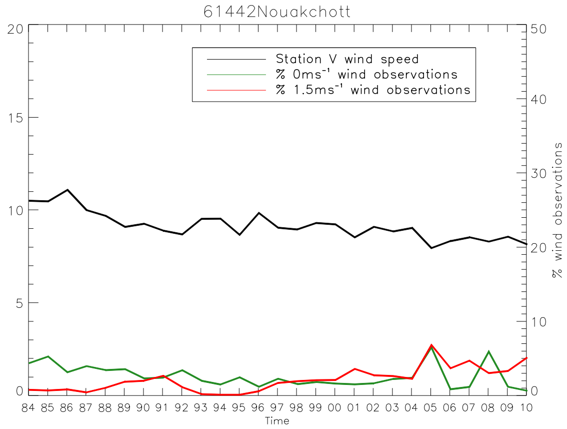

g)

Auxiliary Figure ~~2~~4: Instrument degradation analysis. Time series of mean wind V (black), % of 0 ms^{-1} reports (green) and % of 1.5 ms^{-1} reports (red) for the individual stations a) Agadez, b) Gouré, c) Niamey, d) Gao, e) Tombouctou, f) Nema, and g) Nouakchott. Percentage lines are calculated relative to all observations available for a given year. Trend line values for the green and red lines can be found in Aux. Table 2 and for the black line in Table 1 of the main paper.

Auxiliary Table 2: Instrument degradation trends and correlations

		**Agadez**	**Gouré**	**Niamey**	**Gao**	**Tomb.**	**Nema**	**Nouakchott**
**1**	**0 ms^{-1} trend**	**-52**	***-186***	**-33**	***39***	***112***	***-190***	**49**
**2**	**<1.5 ms^{-1} trend**	***-1228***	***-176***	**-220**	**-2**	**-119**	***99***	***-1909***
**3**	**Correlation: <1.5 ms^{-1}/0 ms^{-1}**	***0.65***	**0.52**	**-0.4**	**-0.29**	**-0.07**	**-0.16**	**-0.02**

Auxiliary Table 2. Rows 1 and 2 contain the 0 ms^{-1} trend (green lines) and <1.5 ms^{-1} trend (red lines) values as plotted in Aux. Figure 3. Row 3 gives the corresponding linear correlation. Statistical significance of trends and correlations at the 95% and 99% levels are denoted in bold and in bold italics, respectively.

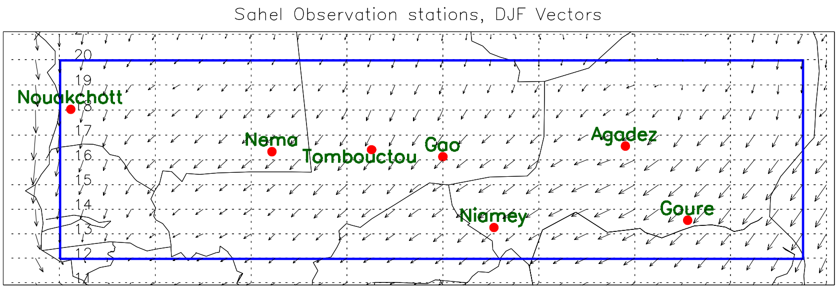
Sahel Observation stations, DJF Vectors
Nouakchott
Nema
Tombouctou
Gao
Agadez
Niamey
Goure

a)

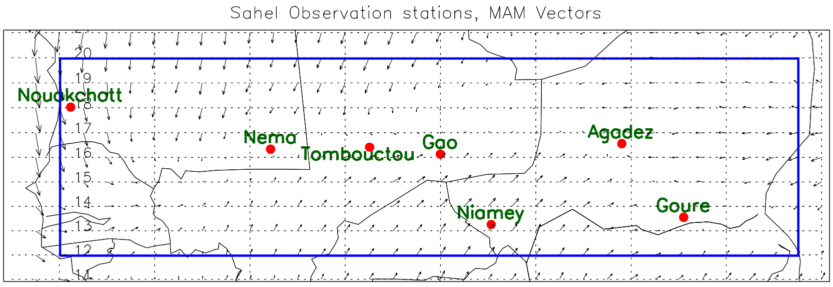
Sahel Observation stations, MAM Vectors
Nouakchott
Nema
Tombouctou
Gao
Agadez
Niamey
Goure

b)

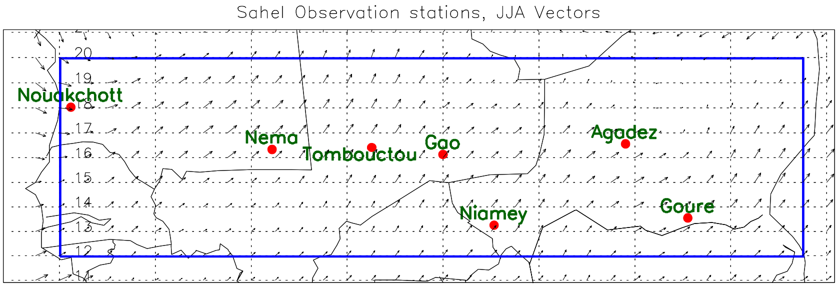
Sahel Observation stations, JJA Vectors
Nouakchott
Nema
Tombouctou
Gao
Agadez
Niamey
Goure

c)

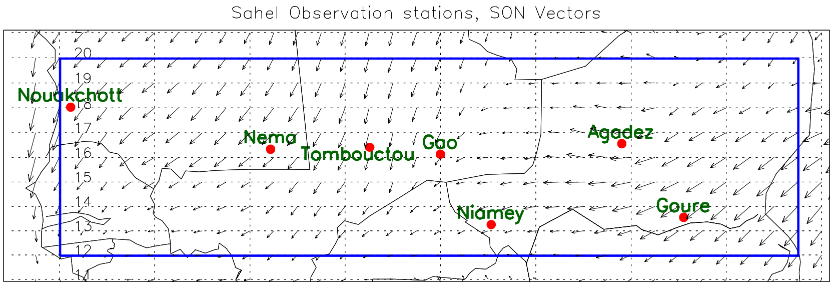
Sahel Observation stations, SON Vectors
Nouakchott
Nema
Tombouctou
Gao
Agadez
Niamey
Goure

d)

Auxiliary Figure ~~3~~5: Seasonality of ERA mean wind vectors. Map of the Sahel with station locations and the ERA-Interim 10 m mean wind vectors for a) DJF, b) MAM, c) JJA, and d) SON similar to Fig. 1 of the main paper. The blue box is the area domain used for averaging ERA 10-m winds in the trend and correlation analysis.

Auxiliary Table 3: Seasonality of ERA mean wind and DUP

		**DJF**	**MAM**	**JJA**	**SON**	**Year**
**1**	**% change in ERA V**	**-7**	**-4**	**2**	***-5***	***-3***
**2**	**% change in ERA DUP**	**-31**	**-5**	**2**	**-27**	**14**
**3**	**ERA V / ERA DUP correlation**	***0.85***	***0.83***	**0.50**	***0.80***	***0.60***
**4**	**ERA V / Obs V correlations**	***0.71***	**0.56**	**-0.07**	**0.53**	**0.56**

Auxiliary Table 3: Seasonality of ERA mean wind and DUP. Relative changes (in %) of ERA-Interim mean wind V and DUP. Rows 1 and 2 are computed in the same way as rows 1–3 in Table 1 in the main text. Statistical significance of trends and correlations at the 95% and 99% levels are denoted in bold and in bold italics, respectively.

Auxiliary Table 4: Correlations of mean wind and observed dust with the NAO index.

		DJF	MAM	JJA	SON	Year
**1**	**V ERA**	***0.77***	**0.31**	**-0.21**	**0.43**	**0.58**
**2**	**V OBS**	**0.5**	**0.14**	**0.43**	**0.28**	**0.46**
**3**	**FDE**	**0.58**	**0.36**	**0.26**	**0.3**	**0.52**

Auxiliary Table 4: Correlations of mean wind and observed dust with the NAO index. Seasonal correlations of ERA-Interim mean wind V (row 1), observation mean wind V (row 2), and observed FDE (row 3) with the seasonal Jones NAO Index (as described in Section 2). Significance of trends and correlations at the 95% and 99% levels are denoted in bold and in bold italics, respectively.

Auxiliary Table 5: Day/night station trends comparison

			**DJF**	**MAM**	**JJA**	**SON**	**Year**
**Absolute trends (ms^{-1} a^{-1})**	**1**	**Day V**	***-0.07***	***-0.05***	***-0.05***	***-0.06***	***-0.06***
	**2**	**Night V**	***-0.09***	***-0.05***	***-0.05***	***-0.04***	***-0.04***
	**3**	**Day DUP**	***-9.5***	***-8.1***	***-5.5***	***-4.9***	***-7***
	**4**	**Night DUP**	***-3.8***	**-2.6**	***-3.8***	***-1.7***	***-3***
**relative change (%)**	**5**	**Day V**	***-30***	***-25***	***-27***	***-36***	***-28***
	**6**	**Night V**	**-59**	***-35***	***-39***	***-53***	***-31***
	**7**	**Day DUP**	***-83***	***-86***	***-87***	***-92***	***-86***
	**8**	**Night DUP**	***-105***	***-91***	***-98***	***-79***	***-97***

Auxiliary Table 5: Day/night station trends comparison. Seasonal absolute and relative trends of day and night data from station averaged observations. Absolute trends in rows 1–4 are calculated as the average change in wind speed per year, while rows 5–8 represent the total change in wind speed during the study period 1984–2010 as a % of the initial value. Significance of trends at the 95% and 99% levels are denoted in bold and in bold italics, respectively.
